# Identification of biological indicators for human exposure toxicology in smart cities based on public health data and deep learning

**DOI:** 10.3389/fpubh.2024.1361901

**Published:** 2024-05-30

**Authors:** Peimao Gao, Guowu Huang, Lu Zhao, Sen Ma

**Affiliations:** ^1^Chongqing General Hospital, Chongqing, China; ^2^School of Public Administration, Sichuan University, Chengdu, China; ^3^People’s Hospital of Fengjie, Chongqing, China

**Keywords:** public health, deep learning, biological indicators, environmental pollution, smart cities

## Abstract

With the acceleration of urbanization, the risk of urban population exposure to environmental pollutants is increasing. Protecting public health is the top priority in the construction of smart cities. The purpose of this study is to propose a method for identifying toxicological biological indicators of human exposure in smart cities based on public health data and deep learning to achieve accurate assessment and management of exposure risks. Initially, the study used a network of sensors within the smart city infrastructure to collect environmental monitoring data, including indicators such as air quality, water quality, and soil pollution. Using public health data, a database containing information on types and concentrations of environmental pollutants has been established. Convolutional neural network was used to recognize the pattern of environmental monitoring data, identify the relationship between different indicators, and build the correlation model between health indicators and environmental indicators. Identify biological indicators associated with environmental pollution exposure through training optimization. Experimental analysis showed that the prediction accuracy of the model reached 93.45%, which could provide decision support for the government and the health sector. In the recognition of the association pattern between respiratory diseases, cardiovascular diseases and environmental exposure factors such as PM2.5 and SO2, the fitting degree between the model and the simulation value reached more than 0.90. The research design model can play a positive role in public health and provide new decision-making ideas for protecting public health.

## 1 Introduction

With the acceleration of urbanization, the risk of urban populations being exposed to environmental pollutants is increasing. Safeguarding public health becomes paramount in the construction of smart cities (1, 2). Factors such as light intensity in living environments, air quality, and water pollution are intricately linked to human health and livelihood. Environmental pollution remains a significant global challenge. Biological indicators are among the measures reflecting the impact of environmental pollution on organisms, providing crucial evidence for assessing environmental pollution risks (3–5). However, the selection and assessment of biological indicators currently rely primarily on empirical methods, lacking systematic and scientific approaches. Some scholars have proposed statistical methods to analyze and predict biological indicators, yet these often necessitate extensive data and complex models, limiting widespread practical application (6, 7). Moreover, other scholars have suggested deep learning-based approaches to predict and classify biological indicators, but these methods typically apply to singular biological indicators or simple data classifications, struggling to address complex multi factor analysis and prediction issues (8, 9). Hence, this study aims to develop a method for identifying biological indicators for urban human exposure toxicology in smart cities based on public health data and convolutional neural networks (CNN), striving for precise assessment and management of exposure indicators and risks. The innovation of the research lies in establishing a database encompassing various environmental pollutants, their types, concentrations, and related information based on public health data. It employs deep learning algorithms to process and analyze environmental and health data. The study comprises four parts: a literature review section outlining the current domestic and international research status, a technical introduction detailing the specific processes of the constructed models and relevant technologies, an experimental section analyzing model performance through designed experiments, and a conclusion section further discussing the results of the experimental analysis and providing prospects for future research.

## 2 Related works

In recent years, with economic development, environmental pollution has significantly affected human daily life and overall well-being. Monitoring and analyzing environmental exposure data play a crucial role in maintaining human health. S. G. Al-Kindi and colleagues explored the impact of air pollution on human health. Through a retrospective statistical analysis of existing literature, they found a close association between cardiovascular practice risk and overall mortality rates with PM2.5 across a range of exposure levels. Measures to reduce cardiovascular risk in response to this association were discussed (10). Wolf et al. found evidence of the health effects of low-level air pollution. They conducted a pooled analysis of individual data from six population-based cohorts in ELAPSE, originating from Sweden, Denmark, the Netherlands, and Germany. The results indicated an association between long-term exposure to air pollutants, even below current limits, and the incidence of stroke and coronary heart disease (11). Lesser et al. evaluated the relationship between pesticide exposure and attention deficit hyperactivity disorder or autism spectrum disorders. A systematic review of existing literature revealed that out of 29 retained studies, 10 reported a significant association between pesticide exposure and these diseases. However, the strength of this association and potential confounding factors varied considerably across different studies (12). Yuchi et al. explored the combined impact of noise and greenery on cognitive impairment symptoms. They investigated the relationship between road distance, air pollution exposure, and the joint effects of noise and greenery on mental disorders. The analysis showed an association between air pollution and the incidence of neurological disorders, while noise exposure did not affect this association (13). Lerchl et al. investigated the impact of intermediate-frequency noise on organ development using exposure analysis on 160 female mice. The results indicated that exposure at 20 kHz, 360 μT did not have adverse effects on tumor development and incidence. However, significant differences in behavioral tests suggested a potentially higher level of alertness in mice (14).

CNN, with its excellent performance, is commonly employed in fields such as image recognition and natural language processing. Zhuge et al. proposed two new methods to automatically and non-invasively differentiate between low-grade gliomas and high-grade gliomas on conventional MRI images using deep CNNs to improve the accuracy of glioma grading. The approach involves initial image correction preprocessing followed by image segmentation using R-CNN and U-Net models. The results indicated that the model achieved a recognition accuracy of 97.2% (15). Wang et al. discovered the crucial role of circular RNAs in human diseases, emphasizing the significance of using them as biomarkers for human disease diagnosis and understanding disease mechanisms. They introduced an efficient computational method based on a combination of multiple sources of information and CNN to predict the association between this biological indicator and diseases. The method demonstrated a prediction accuracy of over 85% and a sensitivity of 88.50% (16). Zhao et al. addressed the time-consuming and expensive challenges of identifying drug-target interactions in new drugs. They proposed a drug-protein pair network based on various drugs and proteins. The network’s edges were associated, and they introduced a framework based on graph CNN for feature extraction of drug-protein pairs’ correlations. Subsequently, a deep neural network was utilized for label prediction (17). Sungheetha, in response to challenges in early identification of diabetic patients using retinal lesion images, proposed the use of CNN for feature extraction. A classification framework using the confusion matrix was applied to identify hard exudates in retinal images. The results indicated that the detection accuracy of this method surpassed traditional detection methods (18). Chen et al. tackled the lack of consideration of prior knowledge of the interested system in fault diagnosis methods using deep learning. They introduced a fault diagnosis method based on graph convolutional networks (GCN). This method combines existing measurement values with prior knowledge, initially diagnosing faults using structural analysis methods and then further diagnosing them using GCN. The results demonstrated a fault diagnosis accuracy exceeding 90% (19).

Based on the literature discussed above, it is evident that CNN is widely used in image recognition, natural language processing, and extensively applied in the medical field. However, existing research has limited exploration of the connection between environmental data and pathological indicators of human exposure, with minimal utilization of computer methods for exploration. In order to achieve more accurate and efficient identification of pathological indicators and provide a healthier and safer urban environment for residents, research has been conducted to build a correlation model between public health indicators and environmental indicators using deep learning and public health data. The aim is to achieve precise assessment and management of exposure risks.

## 3 Construction of a human exposure toxicology biomarker identification model based on public health data and deep learning

The study initially utilized sensor networks in smart city infrastructure to collect environmental monitoring data, laying the foundation for subsequent analysis. Building on a database that includes information on the types and concentrations of environmental pollutants, the research employed deep learning algorithms to process and analyze environmental and health data. Through training optimization, the model identified biological indicators associated with exposure to environmental pollution.

### 3.1 A database based on public health data and environmental monitoring data

**Figure 1:**
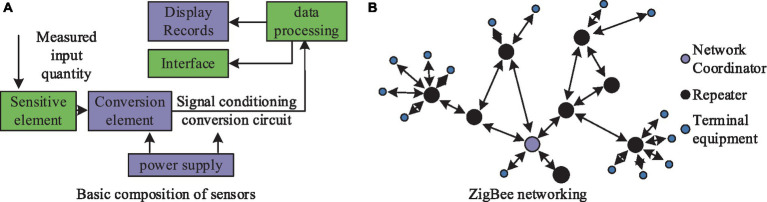
Basic composition of the sensor and the structure of the ZigBee network.

In the model construction phase, the first step involves collecting environmental monitoring data gathered by sensor networks in smart city infrastructure, including indicators such as air quality, water quality, and soil pollution. These data will be used to train and optimize deep learning models to identify biological indicators related to environmental pollution (20–22). The Internet of Things (IoT) primarily contributes to data collection through sensing and identification and is a key data source for IoT. The study utilized wireless communication and set up sensors to monitor environmental data. IoT fulfills the requirements for information transmission, storage, processing, recording, display, and control. The basic components of sensors and the structure of ZigBee networking are illustrated in Figure 1.

Data from the monitoring process include temperature, humidity, light intensity, wind speed, air quality, water pollution, and other factors. Epidemiology, which focuses on disease descriptions in populations, examines physiological indicators of diseases. Epidemiology describes diseases from three perspectives: the temporal distribution, spatial distribution, and distribution among populations. The impact of the environment and climate on diseases is particularly prominent (7, 23, 24). The study collected 200,000 health examination records from a tertiary hospital’s examination center. Initially, deallergization techniques were used to remove personal privacy-related data. Secondly, some health examination data had a high degree of missing feature items, and these data were directly excluded. Finally, 110,000 health examination records were retained as the study dataset. Each record contains over 100 features related to health examinations. The study selected chronic diseases such as fatty liver, hypertension, and diabetes for training the pathological indicator identification model. Eighty percent of the health examination data were used as the training set, and the remaining 20 percent were used as the test set. Various features in public health examination data are numerical, but the values of each feature differ, and each feature’s values are not on the same scale. Due to the lack of precision of sensor equipment, inaccurate calibration and environmental interference, there are errors in the collected environmental data. When these error data are used to train deep learning models, noise and bias will be introduced, which will affect the accuracy and reliability of the model. In the stage of data pre-processing and feature extraction, if the method used is inappropriate or the parameter setting is unreasonable, it will lead to the loss or deformation of data information. In the process of medical data collection and processing in smart cities, a large amount of personal privacy information is involved. If the system has security loopholes or mismanagement, it may lead to the disclosure of patient information, which will bring unnecessary troubles and risks to patients.

For this study, high-precision sensor equipment is selected, and regular calibration and maintenance are carried out to ensure that the collected data is accurate and reliable. At the same time, according to the characteristics and requirements of the data, the appropriate data preprocessing and feature extraction methods are selected to ensure the integrity and effectiveness of the data information. In addition, advanced security technology and management means are adopted to ensure the security and stability of the system and prevent data leakage and illegal access. To eliminate differences in orders of magnitude and dimensional scales among features, the study opted for the Min-max method to normalize environmental monitoring data and physical examination data. The specific calculation method is shown in Equation 1.


(1) $$x^{\prime }=\frac{x-\min\left(x\right)}{\max\left(x\right)-\min\left(x\right)}$$

In Equation 1, $x^{\prime }$ represents the normalized data, and x denotes the numerical values of the features in the physical examination data. In addition to normalizing the data features, the study conducted a simple analysis of various chronic disease markers. In the process of correlation analysis, the distribution of markers is a crucial factor affecting model accuracy. Therefore, the study employed two methods, Binary Relevance (BR) and Label-Powerset (LP), to classify disease data based on labels. The BR method initially divided the original physical examination dataset into mutually independent datasets, each containing corresponding disease types. Subsequently, the LP method combined all labels of samples with multiple labels to create a new label. Since the BR method did not consider the interdependence of labels, the study introduced the Pearson correlation coefficient concept and proposed an association loss function to enhance the interdependence among mutually independent samples in the BR method during model training. The calculation method of the association loss function is shown in Equation 2.


(2) $$CL=\text{loss}+\sum_{i=1}^{l}\alpha_{i}loss_{i}$$

**Figure 2:**
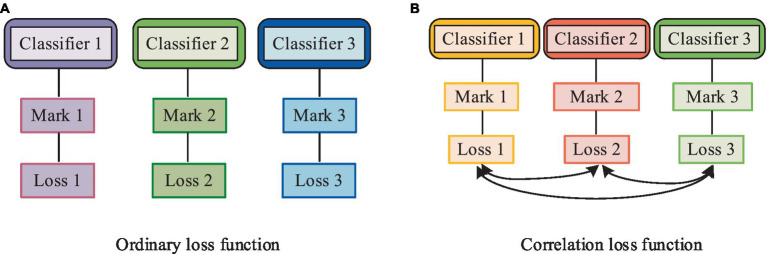
Mechanism of action of the association loss function. **(A)** Ordinary loss function; **(B)** Correlation loss function.

In Equation 2, loss represents the loss learned by the classifier for a single label, and α is the correlation coefficient for different labels. The study used the cross-entropy loss function as the loss function. The mechanism of action of the association loss function is illustrated in Figure 2.

In recent years, Dynamic Factor Analysis (DFA), as a multivariate statistical method for dimensionality reduction, has been designed for time series analysis to reveal the extent to which explanatory variables and common trends influence response variables in multivariate data. This method is particularly relevant to factors related to public health, such as pollution in the environment, especially PM2.5 and photochemical pollution. Leveraging the characteristics of DFA, the study can explore the relationships between different air pollutants and key factors affecting air quality. To associate environmental monitoring data with public health data, the study initially used DFA to preprocess environmental monitoring data, connecting multiple data sources to establish a database. However, seasonal issues in the DFA process can lead to high correlations between indicators, impacting the analysis results. Therefore, the study optimized the time series model as shown in Equation 3.


(3) $$y_{t}=\text{tren}d_{t}+\text{sea}\sin al_{t}+\text{remainde}r_{t}$$

In Equation 3, $y_{t}$ represents the original variables, $\text{tren}d_{t}$ denotes the trend of change, $\text{Seasona}l_{t}$ stands for seasonal factors, and $\text{remainde}r_{t}$ represents the remaining information. By removing the seasonal component, the sum of the trend and the residual information is considered as new observed data. Selecting an appropriate number of common trends and suitable explanatory variables can determine the fitting effect of the observed sequence. Hence, this study employs the Nash-Sutcliffe efficiency coefficient and the Akaike Information Criterion (AIC) for model selection. The efficiency coefficient is used to assess the degree of model fitting, as specifically illustrated in Equation 4.


(4) $$C_{\text{eff}}=\frac{\sum_{t=1}^{T}{\left(Q_{0}^{t}-Q_{m}^{t}\right)}^{2}}{\sum_{t=1}^{T}\left(Q_{0}^{t}-{\bar{Q}}_{0}\right)}$$

In Equation 4, $Q_{0}^{t}$ denotes the observation value at time t, $Q_{m}^{t}$ represents the simulated value at time t, ${\bar{Q}}_{0}$ is the average value of the observed data, and $C_{\text{eff}}$ is an efficiency coefficient ranging between negative infinity and 1. An efficiency coefficient of 1 indicates consistency between the predicted and observed values. The AIC, as a method to measure and compare the goodness of fit of models, where smaller values indicate better model performance. The computation method for AIC is illustrated in Equation 5.


(5) $$AIC=2m^{\prime }-2\ln\left(L\right)$$

**Figure 3:**
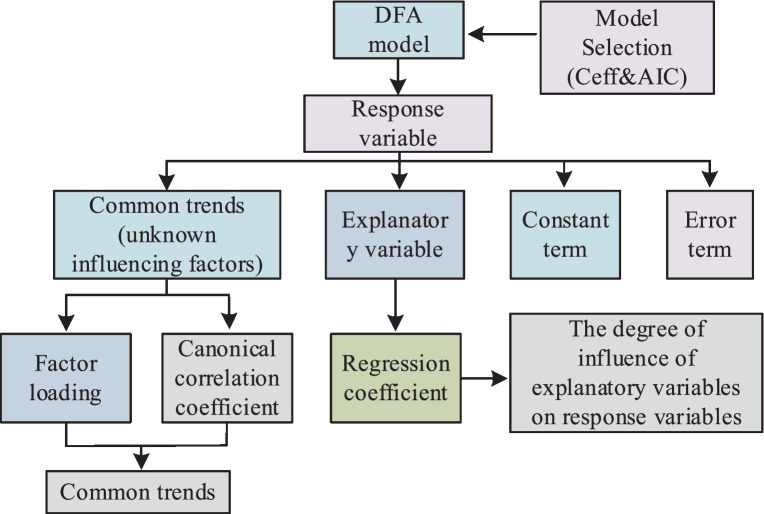
DFA model structure and processing of environmental monitoring data process.

In Equation 5, $m^{\prime }$ represents the number of independent parameters in the model, and L is the value of the likelihood function. The structure of the DFA model and the process for handling environmental monitoring data are depicted in Figure 3.

Considering the above content, the research stores processed and correlated data in a database for subsequent analysis and applications. Additionally, it’s crucial to back up and manage the data to ensure its security and reliability. Through these steps, a database containing information about types of environmental pollutants, their concentrations, etc., can be established and linked with public health data. These datasets will serve as foundational support for subsequent training and optimization of deep learning models.

### 3.2 Smart city toxicology biomarker identification model based on deep learning

**Figure 4:**
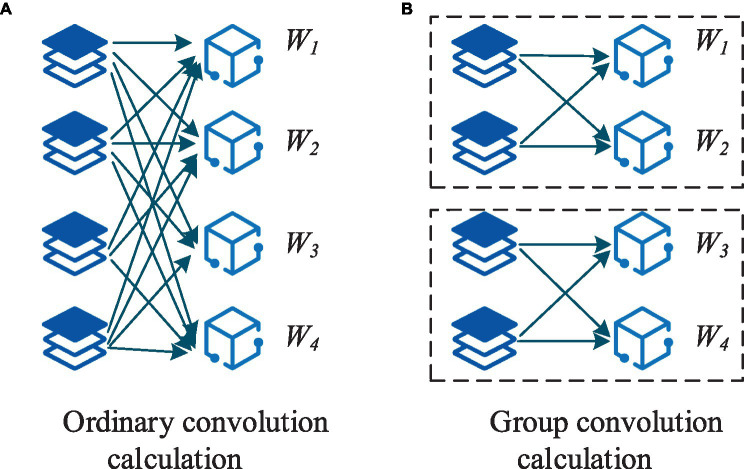
Groups of convolution are calculated. **(A)** Ordinary convoltion calculation; **(B)** Group convolution calculation.

The study utilizes CNN for pattern recognition and correlation analysis of environmental monitoring data. Initially, the collected environmental monitoring data, characterized by spatial and temporal features, undergo preliminary preprocessing. Subsequently, a CNN model is constructed to automatically extract features and recognize patterns within the environmental monitoring data. Training the CNN model helps extract correlated features among different indicators and analyze the degree of correlation and influencing factors. The use of group convolution strategies enhances the sparsity of the network framework, reduces convolutional parameters, accelerates the training speed of the network framework, and effectively increases sparsity to alleviate convolutional redundancy (25–27). The computational approach for group convolution is depicted in Figure 4.

**Figure 5:**
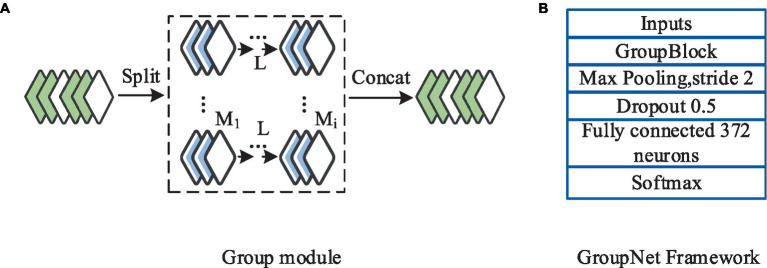
Schematic diagram of the group of convolution frames and modules. **(A)** Group module; **(B)** GroupNet Framework.

The research was inspired by group convolution strategies and designed a novel convolutional module integrating the group convolution approach. This module comprises two parts: regular convolution and clustered convolution. Within the group module, each convolutional layer executes uniform partitioning strategy until the final convolutional layer. Following the group convolution section is the clustered convolution part, employing a 1×1 kernel size. The purpose of the clustered convolutional layers is to compute associations and cluster the features obtained from the group convolution section. The core component of the CNN framework designed in this study is the group module. The entire network architecture consists of six parts: an input layer, the group module, a max-pooling layer, a dropout layer, a fully connected layer, and an output layer. The framework involves a schematic representation of the group convolution and the group module, as depicted in Figure 5.

As shown in Figure 5, each convolutional unit in the group convolution part was configured with 8 convolutional kernels. The clustered convolution part comprises only one 1×1 clustered convolutional layer with 12 convolutional kernels. Following the group module is the max-pooling layer, utilized to reduce computation and extract features. Subsequently, a dropout layer was introduced with a dropout rate set at 0.5, aimed at mitigating overfitting during the model training process (28, 29). The study uses a cross-validation method to determine the optimal dropout rate, and the optimal value can be determined when the performance on the verification set reaches the optimal value. After constant adjustments through training, the study set the dropout rate to 0.5 based on the test results. For learning rate, number of convolution kernel and other parameters, Bayesian optimization is selected to optimize the global parameters and adjust the hyperparameters. In the training process, the posterior distribution of the objective function is constructed to guide the search process, and then the optimal parameter combination is found. The fully connected layer is employed to globally integrate the features extracted from the convolutional layers, with 372 neurons configured. The output layer utilizes the Softmax function as a classifier, computed as per Equation 6.


(6) $$\left\{ \begin{array}{c} Z_{i}=\sum_{k}h_{k}w_{ki}\\ p_{i}=\frac{\exp\left(Z_{i}\right)}{\sum_{j}^{8}\exp\left(Z_{j}\right)} \end{array}\right.$$

In Equation 6, $h_{k}$ represents the neuron nodes activated in the penultimate layer, $w_{ki}$ signifies the weight matrix linking the penultimate layer and the Softmax layer, $Z_{i}$ denotes the input to the Softmax layer, and $p_{i}$ stands for the probability of each category. The research opted for the association loss function to evaluate the training process and optimize the model. Post feature extraction and association analysis of environmental monitoring data, the study employed a recurrent neural network (RNN) to temporally correlate public health data with environmental monitoring data, constructing a model linking health indicators with environmental indices. RNNs are a category of deep network architectures adept at handling sequential data and capturing long-term dependencies between data points. During RNN training, the issue of vanishing or exploding gradients often arises. To address this, the research proposed incorporating Long Short-Term Memory (LSTM) units into the CNN. Based on practical results, the study found that LSTM required extensive parameter tuning, thus opting to utilize Gated Recurrent Units (GRUs) within the LSTM. GRUs consist of two gates: a reset gate and an update gate. The former is calculated as shown in Equation (7).


(7) $$r_{t}=\sigma \left(W^{r}x_{t}+U^{\prime }h_{t-1}\right)$$

In Equation 7, $h_{t-1}$ represents the previous moment’s state information, $x_{t}$ is the current time input, $U^{r}$ is the weight matrix in the reset gate, $W^{r}$ is the output value of the hidden layer in the reset gate from the previous time step, and σ is the sigmoid function. The calculation of the update gate is given by Equation 8.


(8) $$z_{t}=\sigma \left(W^{z}x_{t}+U^{z}h_{t-1}\right)$$

In Equation 8, $U^{z}$ and $W^{z}$ are the weight matrix and the output value of the hidden layer in the update gate, respectively. The information from the previous time step and the current input are passed through an activation function to be within the range of [−1, 1], and then the reset gate resets useful information. The update gate simultaneously performs information forgetting and selection for storage. The candidate hidden layer memorizes new information at the current time step, and the reset gate controls how much of the previous information to retain. The output information of the hidden layer is shown in Equation 9.


(9) $$h_{t}=z_{t}⊙h_{t-1}+\left(1-z_{t}\right){\tilde{h}}_{t}$$

In Equation 9, $z_{t}⊙h_{t-1}$ represents forgetting unimportant information from $h_{t-1}$, $z_{t}$T is the discarded information, $1-z_{t}$ compensates for the discarded part using the weights corresponding to the current input features, and ${\tilde{h}}_{t}$ is the information at the current time step. The definition of the GRU is given by Equation 10.


(10) $$net_{h}^{t}=Ux_{t}+Ws_{t-1}+b_{h}$$

**Figure 6:**
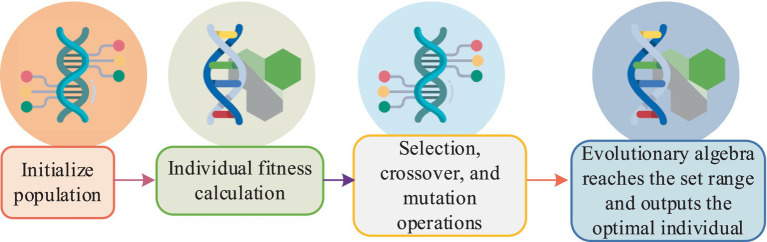
Flow diagram of the genetic algorithm.

In Equation 10, $x_{t}$ is the input at time t, $net_{h}^{t}$ represents the weighted sum of the hidden layer activation before activation at time t, U is the input weight matrix at time t, and W is the recurrent matrix for the hidden layer at time t. During the training of deep RNNs, the adaptive moment estimation (Adam) training algorithm is used for optimizing neural networks. The advantages of this algorithm include efficient computation, minimal tuning, low memory requirements, fast convergence, and invariance to gradient diagonal scaling. To achieve dimensionality reduction and optimization in the process of nonlinear feature extraction in multivariate data, the study combines genetic algorithms (GA) with GRU. The genetic algorithm’s process is illustrated in Figure 6.

The research employs the mean squared error (MSE) between predicted values and actual values as the fitness function, as shown in Equation 11.


(11) $$E\left(y_{i}\right)=\frac{1}{n}\sum_{i=1}^{n}{\left(y_{i}^{\prime }-y_{i}\right)}^{2}$$

In Equation 11, $y_{i}^{\prime }$ and $y_{i}$ are the actual measured values and predicted output values for the ith individual, and n is the number of samples in the training set. The calculation method for the total population fitness is given by Equation 12.


(12) $$f=\sum_{i=1}^{n}E\left(y_{i}\right)$$

In accordance with the selection strategy, the study uses the roulette wheel selection method based on the fitness ratio to design the genetic operators used in the genetic algorithm. The definition of the selection operator is given by Equation 13.


(13) $$P_{s}=\frac{1/E\left(y_{i}\right)}{\sum_{1}^{num}1/E\left(y_{i}\right)}$$

In Equation 13, $E\left(y_{i}\right)$ represents the individual fitness function, and num is the population size. For individuals encoded as real numbers, real crossover is employed during the crossover operation to exchange individual positions. For elements at corresponding positions in an individual, mutation operations are performed according to the mutation probability to generate new individuals. The position mutation operator is defined as shown in Equation 14.


(14) $$H_{z^{\prime }k}=H_{\text{min}}+\left(H_{\text{max}}-H_{\text{min}}\right)*\text{rand}\left[0,1\right]\;\text{rand}\left[0,1\right]\geq P_{v}$$

**Figure 7:**
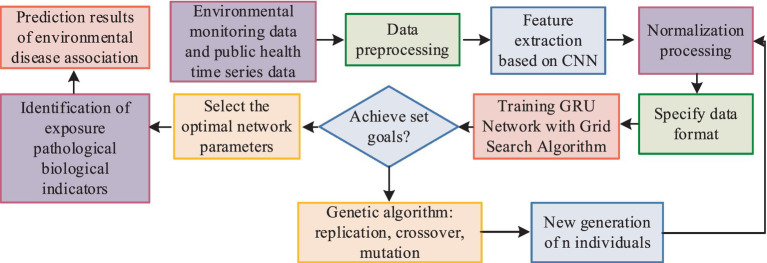
Model training and prediction process.

In Equation 14, $H_{\text{min}}$ and $H_{\text{max}}$ represent the minimum and maximum values of the elements, respectively, and $P_{v}$ represents the mutation probability. When $\text{rand}\left[0,1\right]<P_{v}$, random numbers continue to be generated until they are not less than the mutation probability. The optimized pathological index model is shown in Equation 15.


(15) $$y_{t}^{\prime }=F\left(y_{1},y_{2},\ldots ,y_{t-1},x_{1},x_{2},\ldots ,x_{t-1}\right)$$

in Equation 15, $\left(x_{1},x_{2},\ldots ,x_{t-1}\right)$ represents the time series of environmental monitoring data and physical examination data, $y_{1},y_{2},\ldots ,y_{t-1}$ is the historical series of disease changes in public health data every day, and $y_{t}^{\prime }$ is the predictive result of the epidemic in the urban population. The training and prediction process of the model is illustrated in Figure 7.

By obtaining the correlation results between environmental exposure pollution and citizen health through the model, pathological indicators can be identified. This enables timely understanding and prediction of the occurrence of urban public health events through monitoring environmental data. The predicted results can be used for timely management and assessment of public health, preventing the widespread occurrence of diseases. For example, when environmental monitoring data shows a high concentration of harmful substances, the public health management system can issue timely warnings and provide corresponding health protection measures.

To more fully assess the risks associated with the proposed environmental toxin monitoring system via wireless signals, the study first assessed the direct effects of electromagnetic radiation that may be generated during wireless signal transmission on the human body. Although studies have shown that low-power wireless signals have relatively small effects on the human body, there may still be some health risks under long-term, sustained exposure. Therefore, the study strictly limits the transmitting power of the monitoring system, and increases the shielding device in practical applications to reduce the potential harm of electromagnetic radiation to the human body.

In addition, in order to mitigate the main effects of Radio Frequency Radiation (RFR) and the synergies that may occur on the human exposure pathway, research is conducted to reduce energy consumption and electromagnetic radiation during data transmission by optimizing the wireless communication protocol of the system. In addition, when deploying monitoring equipment, avoid deploying too much equipment in crowded areas or sensitive areas. Finally, establish a long-term and continuous monitoring mechanism to detect and deal with possible environmental problems in a timely manner to protect the health of the public. In this way, the risks associated with environmental toxin monitoring system through wireless signal can be minimized, and strong support can be provided for urban public health management.

## 4 Performance analysis of the smart city toxicology index identification model based on deep learning

To evaluate the performance of the smart city toxicology index identification model, various evaluation metrics, including accuracy, recall, F1 score, etc., were employed for experimental analysis. PM2.5 has a significant public health impact, and long-term exposure to high concentrations of PM2.5 can increase a variety of health risks, such as heart disease, lung cancer, and respiratory diseases. Therefore, monitoring PM2.5 is essential for assessing environmental quality and its impact on citizens’ health (30). Secondly, PM2.5 comes from a wide range of sources, including natural factors such as volcanic eruptions and forest fires, as well as human factors such as industrial emissions and traffic exhaust. Therefore, the change of PM2.5 concentration is closely related to a variety of environmental factors, such as meteorological conditions, landforms, and urban planning (31). Therefore, through monitoring and analysis of PM2.5, we can gain an in-depth understanding of environmental conditions and the impact of environmental factors on the occurrence and development of diseases. And because the impact of PM2.5 on human health has been widely studied and recognized, its monitoring data is relatively rich and reliable. Using this data in the process of model training and optimization has advantages (32). In summary, as one of the important indicators in environmental monitoring data, PM2.5’s unique nature and influence enable it to effectively represent environmental monitoring data to explore the relationship between environmental indicators and disease indicators. Therefore, the performance of the model designed by the study was analyzed with the typical environmental data of PM2.5.

### 4.1 Parameter adjustment analysis

**Figure 8:**
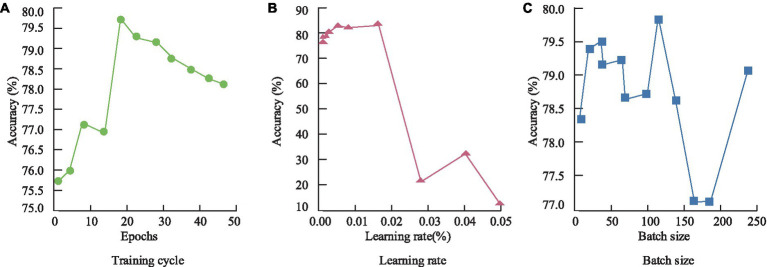
Training period, learning rate and batch size setting rationality experiment of the model. **(A)** Training cycle; **(B)** Learning rate; **(C)** Batch Size.

Training cycles, learning rates, and batch sizes are key factors influencing the performance of neural network frameworks. The study used the following hyperparameters: learning rate of 0.02, batch size of 128, and training cycles of 20. Accuracy was introduced as an evaluation metric to test the rationality of the hyperparameter settings. The experimental results are shown in Figure 8.

**Figure 9:**
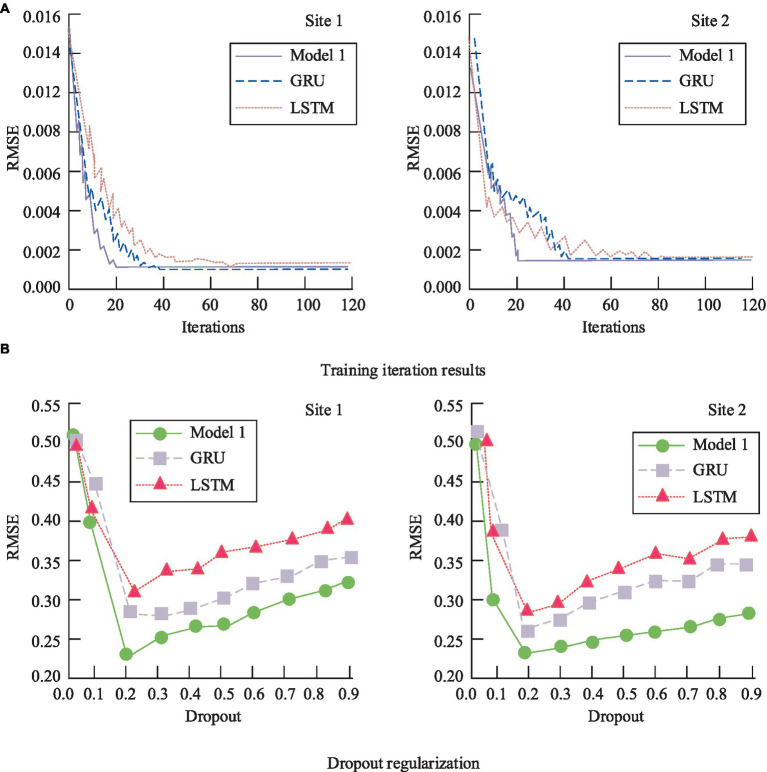
Performance comparison between the model and the single GRU, LSTM. **(A)** Training iteration results; **(B)** Dropout regularization.

From Figure 8A, it can be observed that, overall, accuracy increases with the number of training cycles. However, when the training cycles exceed 20, the model’s accuracy begins to decline, likely due to over-fitting. As shown in Figure 8B, 12 groups of different learning rates were set in the study. From the experimental results, when the learning rate was small, the accuracy of the model was basically the same, and the difference was not large. When the learning rate exceeds 0.02, the accuracy of the model shows a sudden decline. Therefore, the study chooses the value with the highest accuracy, i.e., 0.02, to set the learning rate of the model. Figure 8C indicates a similar relationship between batch size and accuracy as training cycles, with a decline in accuracy when the batch size exceeds 128. Overall, the selected combination of hyper parameters allows the model to achieve optimal performance. For deep neural networks with a large number of parameters, overfitting is a serious issue. Therefore, the study employed dropout regularization during training, randomly dropping some neuron units from the neural network. To further determine the optimal regularization values, the model was tested at two different sites. Additionally, to verify the convergence performance of the constructed model, it was compared with individual GRU and LSTM models, as shown in Figure 9.

According to Figure 9A, it can be observed that after 20 iterations, the RMSE value of Model 1 gradually stabilizes and reaches its minimum. In contrast, both GRU and CNN require more training iterations than Model 1. Figure 9B reveals that with a regularization parameter of 0.2, Model 1 achieves the lowest RMSE value, specifically 0.20, whereas the RMSE values for the standalone GRU and the improved Model 1 are 0.23. The enhanced Model 1 demonstrates superior predictive performance.

### 4.2 Results and analysis of input feature correlation

After conducting a correlation analysis of the model, statistical results of the internal correlations within environmental monitoring data were obtained. Additionally, pathology indicators were employed to identify relationships between diseases and environmental exposure pollution, yielding corresponding correlation results. The study initially selected five clinical indicators related to cardiovascular and respiratory diseases for training and calculation, in relation to air PM2.5. The specific results are presented in Table 1, where respiratory indicators include Cytokeratin 19 fragment (CYFAR21-1) and Neuron-Specific Enolase (NSE), and cardiovascular indicators consist of Creatine Kinase MB (CKMB), Lactate Dehydrogenase (LDH), and C-reactive Protein (CRP).

**Table 1:** Results of association between air index data and respiratory and cardiovascular disease indicators.

Project		First quarter	The second quarter	The third quarter	The fourth quarter	Correlation coefficient
CKMB	Total number of cases	3,055	2,460	3,484	3,585	0.435
	Number of positive cases	283	270	210	241	
	The number of cases associated with PM2.5	23	17	12	18	
	Related cases with PM2.5 (%)	8.12	6.30	5.71	7.47	
LDH	Total number of cases	2,845	3,081	4,215	3,284	0.254
	Number of positive cases	674	589	592	584	
	The number of cases associated with PM2.5	55	35	31	46	
	Related cases with PM2.5 (%)	8.17	5.94	5.24	7.88	
CRP	Total number of cases	9,675	6,842	9,485	8,641	0.481
	Number of positive cases	3,845	2,575	4,082	3,028	
	The number of cases associated with PM2.5	280	88	135	226	
	Related cases with PM2.5 (%)	7.28	3.42	3.30	7.46	
CYFAR21-1	Total number of cases	4,851	5,254	5,580	4,251	−0.597
	Number of positive cases	668	1,548	1,589	621	
	The number of cases associated with PM2.5	59	95	100	52	
	Related cases with PM2.5 (%)	8.83	6.14	6.92	8.37	
NSE	Total number of cases	3,055	3,515	2,544	2,612	0.062
	Number of positive cases	311	432	189	205	
	The number of cases associated with PM2.5	–	–	–	–	
	Related cases with PM2.5 (%)	–	–	–	–	

**Figure 10:**
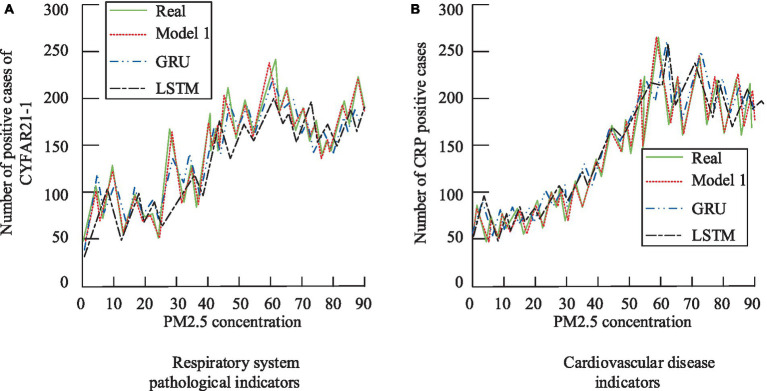
The pathology results obtained from the model prediction are compared with the simulation results. **(A)** Respiratory system pathological indicators; **(B)** Cardiovascular disease indicators.

The pathological identification and correlation analysis results are summarized in Table 1. Notably, CYFAR21-1 in the respiratory system exhibits a negative correlation, while NSE shows no significant correlation with PM2.5. Conversely, the three cardiovascular indicators are significantly correlated with PM2.5 exposure. To further validate the model’s performance, the study compared the predicted pathology results with simulation results, as depicted in Figure 10.

From Figure 10, it is evident that the trend of the model’s predicted values aligns closely with the simulation values, with an average fitting degree of 0.90. Specifically, Figure 10A shows a fitting degree of 0.92 for respiratory pathology indicators, and Figure 10B indicates a fitting degree of 0.88 for cardiovascular diseases. The comprehensive data confirms the model’s excellent performance. To conduct a more comprehensive evaluation, the study compared the constructed model with advanced models from existing research. ROC curves were introduced, and the area under the curve (AUC) was used as one of the evaluation metrics, along with accuracy, precision, recall, and F1 score. The compared models include an environmental pollution and pathology indicator correlation prediction model based on deep matrix decomposition (Model 2), a heterogeneous network-based correlation prediction model (Model 3), and a prediction model based on time series theory and gray system (Model 4). The specific results are presented in Table 2.

**Table 2:** Performance comparison results of each model.

Project	AUC	Accuracy (%)	Precision	Recall	F1
Model 1	0.91	93.45	0.93	0.91	0.92
Model 2	0.83	84.32	0.84	0.81	0.80
Model 3	0.89	90.02	0.90	0.87	0.88
Model 4	0.85	86.94	0.86	0.84	0.83

According to Table 2, the accuracy, precision, recall, and F1 score of Model 1 were 93.45%, 0.93, 0.91, and 0.92, respectively. In comparison to the other three models, Model 1 exhibited significantly superior performance across all metrics. The comprehensive data in Table 2 indicates that Model 1 possesses excellent predictive capabilities.

### 4.3 Analysis of model application based on deep learning and public health data

**Figure 11:**
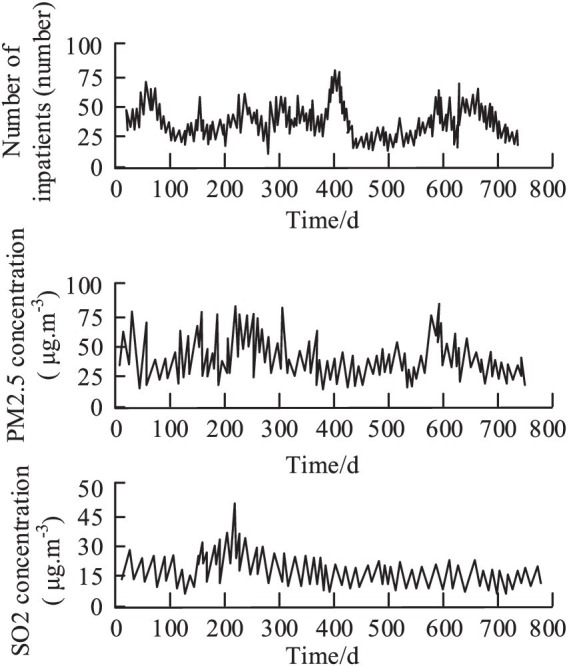
respiratory diseases, PM2.5 and SO2 over time.

To assess the practical application of the constructed models, the study examined the identification of respiratory disease pathologic indicators and the prediction of disease correlations using public health data and environmental monitoring data from four different cities. The temporal variation of respiratory diseases in City A in relation to PM2.5 and SO2 is illustrated in Figure 11.

**Figure 12:**
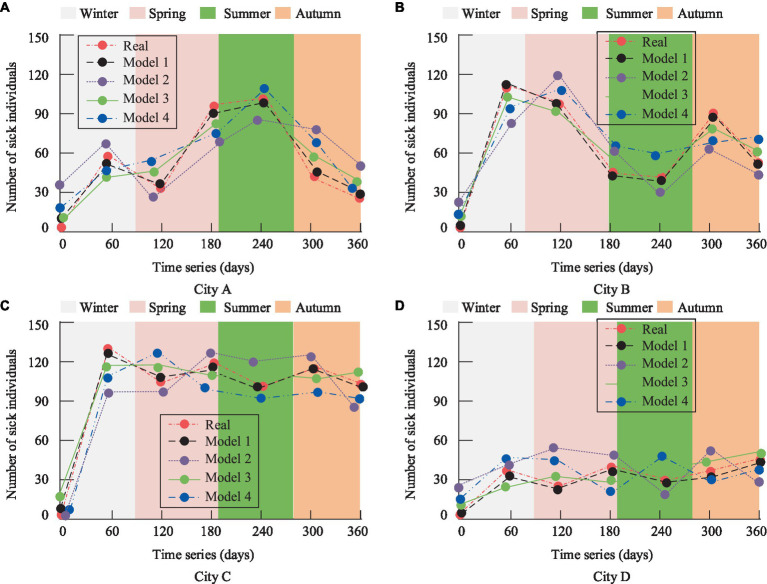
Results of association prediction accuracy of respiratory diseases in the four cities. **(A)** City A; **(B)** City B; **(C)** City C; **(D)** City D.

As evident from Figure 11, PM2.5 exhibits considerable dispersion, with fluctuating concentration values. Meanwhile, the number of respiratory disease cases remains relatively stable. Structurally, respiratory diseases, PM2.5, and SO2 data show a pattern of intense fluctuations in the front followed by a stable distribution. The changing accuracy of respiratory disease correlation predictions over time in the four cities is depicted in Figure 12.

Figure 12A reveals that City A experiences the highest incidence of diseases during the summer and relatively fewer cases in the autumn. Figure 12B shows an increase in residents’ cases in City B during the spring and autumn seasons. City C, an industrial city with consistently poor air quality, has the highest number of cases among the four cities, as shown in Figure 12C. Lastly, Figure 12D indicates that City D, a subtropical coastal tourist city, has relatively fewer respiratory disease cases. Overall, Figure 12 suggests that the predictions of Model 1 closely align with the actual trends, achieving an accuracy of 0.93. While the other three models also exhibit similar trends, they deviate significantly, with fitting accuracies all below 0.90. To comprehensively compare the performance of each model, the study introduced RMSE, MAE, and the Index of Agreement (IA) as evaluation metrics. The specific results are presented in Table 3.

**Table 3:** comparative results of prediction performance of the four models in four different cities.

Project		Training time (h)	RMSE	MAE	IA
A	Model 1	1.00	10.51	8.45	0.94
	Model 2	2.94	19.05	19.45	0.80
	Model 3	1.51	13.54	11.88	0.88
	Model 4	2.05	16.78	15.97	0.85
B	Model 1	1.01	10.55	8.05	0.96
	Model 2	3.00	20.82	19.02	0.79
	Model 3	1.38	13.74	12.04	0.91
	Model 4	2.06	16.45	16.34	0.82
C	Model 1	1.11	10.61	8.16	0.95
	Model 2	3.13	20.45	20.84	0.81
	Model 3	1.69	13.94	12.04	0.90
	Model 4	2.38	15.98	16.98	0.83
D	Model 1	1.05	10.54	7.95	0.93
	Model 2	3.48	20.36	20.84	0.78
	Model 3	1.94	14.56	13.78	0.89
	Model 4	2.49	16.02	17.02	0.84

Table 3 reveals that Model 1 holds a substantial advantage in the numerical values of the three performance evaluation metrics. The IA value of Model 1 exceeds 0.95, indicating superior temporal prediction capabilities. Moreover, the RMSE and MAE values of Model 1 are significantly lower than those of the other three models, at 10.55 and 8.15, respectively. In conclusion, the constructed exposure toxicology biomarker identification model, through recognizing the correlation patterns between environmental exposure data and pathological biomarkers, achieves accurate and efficient disease prediction for smart cities. This provides robust scientific evidence for disease control management departments, aiding them in timely assessments and decision-making.

## 5 Conclusion

The environmental issues are gradually worsening, posing serious risks to the physical and mental health of individuals. In response to this, a new model has been developed based on deep learning and public health data. The aim is to identify the correlation between environmental monitoring data and toxicological indicators, and simultaneously predict public diseases through detection data. The results indicate that the model’s training performance is optimal with a learning rate of 0.02, a batch size of 128, and a training period of 20 cycles. The model, when applied to recognize patterns of correlation between respiratory diseases, cardiovascular diseases, and environmental exposure factors such as PM2.5 and SO2, achieved a fitting degree of above 0.90 with simulated values. Model 1 demonstrated an accuracy, precision, recall, and F1 score of 93.45%, 0.93, 0.91, and 0.92, respectively. In comparison to other models, it exhibited higher fitting accuracy and performance indicators. Model 1’s IA value exceeded 0.95, indicating superior temporal prediction capability. Additionally, both the RMSE and MAE values of Model 1 were significantly lower than the other three models, measuring 10.55 and 8.15, respectively. This suggests that the model can provide a more robust scientific basis for health control management departments, aiding them in timely assessments and decision-making. The current study only explores public health and environmental monitoring data, potentially facing issues of incomplete or low-quality data. Future research could consider integrating knowledge from other relevant fields, such as Geographic Information Systems, to offer more specific and refined recommendations for public health management.

## Data availability statement

The original contributions presented in the study are included in the article/supplementary material, further inquiries can be directed to the corresponding author.

## Author contributions

PG: Investigation, Writing – original draft. GH: Conceptualization, Investigation, Writing – original draft. LZ: Investigation, Writing – original draft. SM: Conceptualization, Supervision, Writing – review & editing.
